# Risk factors for cardiovascular disease in women with preeclampsia and intrauterine growth restriction

**DOI:** 10.12669/pjms.40.9.8184

**Published:** 2024-10

**Authors:** Fehmida Memon, Samia Aijaz, Mahreen Bhatti, Naheed Sheikh

**Affiliations:** 1 Dr. Fehmida Memon, FCPS Associate Professor, Liaquat University of Medical and Health Sciences, Hyderabad, Sindh, Pakistan; 2 Dr. Samia Aijaz, FCPS Consultant Urogynaecologist, Liaquat University of Medical and Health Sciences, Hyderabad, Sindh, Pakistan; 3 Dr. Mahreen Bhatti M. Phil Biochemistry, Army Medical College, National University of Medical Sciences, Rawalpindi, Pakistan; 4 Dr. Naheed Sheikh, FCPS, Professor and Head of Department of Gynecology/Obstetrics, Liaquat University of Medical and Health Sciences, Hyderabad, Sindh, Pakistan

**Keywords:** Risk Factors, Cardiovascular Diseases, Women, Pregnancy, Preeclampsia, Intrauterine Growth Retardation

## Abstract

**Objective:**

To identify the risk factors for cardiovascular disease in women with pre-eclampsia and intrauterine growth retardation.

**Methods:**

This cross sectional study was conducted in Department of Gynecology and Obstetrics, Liaquat University Hospital, Hyderabad, from April 2022 to October 2022. Patients with history of intrauterine growth retardation or preeclampsia after 20 weeks of gestation, age more than 18 years were included in this study. Patients were assessed for cardiovascular risk factors during pregnancy.

**Results:**

The average age and gestational age were almost equal in preeclampsia and IUGR patients, (p≤0.050). The primparous were higher in preeclampsia than IUGR, n=286 (73.5%) and n=80 (52.3%), respectively, (p=0.000). The average birth weight of IUGR was lower than preeclampsia patients, 925.19±6.35 gram and 1324.76±10.19 gram, respectively, (p=0.000). The average systolic and diastolic blood pressure of IUGR patients was less than preeclampsia patients, (p=0.000). But, the chronic hypertension was higher in preeclampsia patients n=99 (25.4%) as compare to IUGR n=13 (8.5%) patients, (p=0.000). The average cholesterol level in IUGR was 5.52±0.58(mmol/L) versus preeclampsia 5.34±1.01(mmol/L), (p=0.043). The average triglycerides was almost equal in both the groups, (p=0.924). The mean Lp(a) in preeclampsia patients was 177.15±20.15(mg/L) versus 202.94±24.83 (mg/L), (p=0.000).

**Conclusion:**

Findings of this study help conclude that women with known history of IUGR or preeclampsia must be screened for possible cardiovascular risk factors and treated for these risk factors in order to avoid future mortality and morbidity associated with cardiovascular diseases.

## INTRODUCTION

One of the leading causes of maternal mortality and morbidity is preeclampsia. It is characterized clinically in terms of severe hypertension, proteinuria which occurring after 20 weeks of gestation. Preeclampsia when becomes severe results in the development of HELLP (hemolysis, elevated liver enzymes and low platelet counts) syndrome.^1^ One of the major complications of preeclampsia or HELLP syndrome is the development of intrauterine growth retardation. Exact pathophysiology of preeclampsia is still unclear. As per the current theories, the main reason is the insufficient placentation which results formation of blood borne substances which when go into maternal circulation result in more known pathophysiological events such as endothelial dysfunction. The endothelial dysfunction in turn causes maternal syndrome i.e. capillary leakage, vasospasm, activation of coagulation cascade, reduced perfusion of the end organs and faulty fibrinolysis. The deteriorated placental function causes intrauterine growth retardation in fetus.^2^ The pathological lesions in preeclampsia are classified as acute atherosis, thrombosis and fibrin deposits.^3^ The similar characteristics of these lesions to those of atherosclerosis have led to believe that underlying mechanism or pathway is common. The common features include insulin resistance, hypercoagulability and dyslipidemia.^4^ Some literature also suggests that women having history of preeclampsia or intrauterine growth retardation of the fetus have higher risk for developing hypertension and cardiovascular diseases later in life.^5^

The link between preeclampsia, IUGR, and cardiovascular diseases suggests maternal risk factors are the major cause. Lipoprotein (a) in concentration above 300mg/dl has been seen to be independently associated with the development of atherosclerosis in the future by facilitating the lipid delivery to endothelial sites of lesion.^6^ Past studies have shown a direct or indirect link between lipoprotein (a) and IUGR and preeclampsia.^7^ The current study focuses on the relationship of intrauterine growth retardation and preeclampsia with risk factors of cardiovascular diseases such as hypertension, raised BMI, low HDL cholesterol, high cholesterol and triglycerides, insulin resistance and raised concentrations of lipoprotein (a).

## METHODS

This study was conducted in department of gynecology and obstetrics, Liaquat University Hospital, Hyderabad from April 2022 to October 2022. Patients with history of intrauterine growth retardation or preeclampsia more than 20 weeks of gestation, or chronic hypertension were included in this study.

### Inclusion & Exclusion Criteria:

Women who had history of major cardiovascular event were excluded from study. Patients diagnosed with pre-ecalampsia and IUGR on antenatal visit and met the inclusion criteria were assessed for cardiovascular risk factors.

### Ethical Approval:

It was taken from the ethical review committee of Liaquat University. Informed consent was taken from the patients before enrollment. Data was collected on predesigned questionnaire.Ref. LUMHS/REC/-83 dated April 13, 2022.

Preeclampsia was defined in terms of presence of hypertension after 20 weeks gestation accompanied by proteinuria >300mg/24Hr or evidence of maternal acute kidney injury, liver dysfunction, neurological features, hemolysis or thrombocytopenia or fetal growth restriction diagnosed on Doppler ultrasound at antenatal checkup. HELLP syndrome was defined in terms of low platelet counts (<100×10^9^/L), high lactate dehydrogenase (>600U/L), high amino aspartate transferase concentration (>70U/L).^2^,^3^ Intrauterine growth retardation was described as low birth weight below the 5^th^ percentile of the normal population diagnosed on doppler ultrasound at antenatal checkup. Variables such as BMI, blood pressure on admission, plasma insulin and glucose, plasma fasting cholesterol, LDL cholesterol, triglycerides, HDL cholesterol and lipoprotein (a) were examined in each patient.

### Statistical Analysis:

Data thus collected was subjected to statistical analysis using the computer software SPSS version 23. Mean and standard deviation was calculated for quantitative variables and frequency and percentage was calculated for qualitative variables. Chi square test was applied to assess the association of outcome variables and effect modifiers. P value of less than or equal to 0.05 was taken as statistically significant.

## RESULTS

Total number of obstetric admissions were 6348, out of that 542 patients were our study participants, in which n=389 (71.8%) were preeclampsia and n=153 (28.2%) patients reported as IUGR. The average age, and gestational age were almost equal in preeclampsia and IUGR patients, (p≥0.050). The prim parous were higher in preeclampsia than IUGR, n=286 (73.5%) and n=80 (52.3%), respectively, (p=0.000). The average birth weight of IUGR was lower than preeclampsia patients, 925.19±6.35 gram and 1324.76±10.19 gram, respectively, (p=0.000). Fasting blood sugar and serum fasting insulin levels in both the groups were almost equal, (p≥0.050), (Table-I).

**Table-I:** Baseline and obstetrical variable of the study groups.

Variable	Group		P-value

	Preeclampsia n=389 (71.8%)	IUGR n=153 (28.2%)	
***Age (years)***			
Mean±S.D	28.29±4.45	27.73±4.21	0.179
***Parity***			
Prim parous	n=286 (73.5%)	n=80 (52.3%)	0.000
Multiparous	n=103 (26.5%)	n=73 (47.7%)
***Gestational age (days)***			
Mean±S.D	214.29±11.15	214.64±9.75	0.735
***Birth weight (g)***			
Mean±S.D	1324.76±10.19	925.19±6.35	0.000
IUGR	n=209 (53.7%)	n=153 (100%)	0.000
HELLP-syndrome	n=221 (56.8%)	n=0 (0%)	0.000

The average systolic and diastolic blood pressure of IUGR patients was less than preeclampsia patients, (p=0.000). But the chronic hypertension was higher in preeclampsia patients n=99 (25.4%) as compare to IUGR n=13 (8.5%) patients, (p=0.000), (Table-I).

The average cholesterol level in IUGR was 5.52±0.58(mmol/L) versus preeclampsia 5.34±1.01(mmol/L), (p=0.043). The average triglycerides was almost equal in both the groups, (p=0.924). The mean Lp(a) in preeclampsia patients was 177.15±20.15(mg/L) versus 202.94±24.83 (mg/L), (p=0.000), (Table-III).

**Table-II:** Risk factors for cardiovascular disease of the study groups

Risk factor	Group		P-value

	Preeclampsia n=389 (71.8%)	IUGR n=153 (28.2%)	
***Systolic blood pressure (mm Hg)***			
Mean±S.D	130.26±5.05	124.99±2.15	0.000
***Diastolic blood pressure (mm Hg)***			
Mean±S.D	85.07±3.15	77.90±3.09	0.000
***BMI (kg/m^2^)***			
Mean±S.D	25.29±2.22	25.63±2.38	0.120
Chronic hypertension	n=99 (25.4%)	n=13 (8.5%)	0.000

**Table-III:** Biochemical risk factors for cardiovascular disease of the study groups.

Risk factor	Group		P-value

	Preeclampsia n=389 (71.8%)	IUGR n=153 (28.2%)	
***Cholesterol (mmol/L)***			
Mean±S.D	5.34±1.01	5.52±0.58	0.043
***HDL-Cholesterol (mmol/L)***			
Mean±S.D	1.50±0.80	1.32±0.55	0.017
***Triglycerides (mmol/L)***			
Mean±S.D	1.19±0.32	1.20±0.38	0.924
***Lp(a) (mg/L)***			
Mean±S.D	177.15±20.15	202.94±24.83	0.000
***p(a) > 300 mg/L (%)***			
Mean±S.D	33.25±3.69	35.54 ± 4.89	0.000

The risk factors for cardiovascular disease after exclusion of women with the potential confounders, chronic hypertension or a BMI > 30. It was seen that n=21 (12.5%) patients were IUGR and n=147 (87.5%) patients were preeclampsia. The mean systolic and diastolic blood pressure was less in IUGR patients versus preeclampsia patients, (p=0.000). While, the cholesterol, HDL-cholesterol, triglycerides were almost equal in both the groups, (p≥0.050), (Table-IV).

**Table-IV:** Risk factors for cardiovascular disease after exclusion of women with the potential confounders, chronic hypertension or a BMI > 30.

Risk factor	Group		P-value

	Preeclampsia n=147 (87.5%)	IUGR n=21 (12.5%)	
***Systolic blood pressure***			
Mean±S.D	128.01±5.93	122.42±6.45	0.000
***Diastolic blood pressure***			
Mean±S.D	85.58±5.94	77.09±1.97	0.000
***Cholesterol (mmol/L)***			
Mean±S.D	5.14±1.31	5.15±1.36	0.952
***HDL-Cholesterol (mmol/L)***			
Mean±S.D	1.44±0.61	1.38±0.65	0.621
***Triglycerides (mmol/L)***			
Mean±S.D	1.01±0.21	0.94±0.23	0.183
Fasting blood sugar	105.72±4.78	108.32±4.32	0.169
Serum fasting insulin	22.54±3.39	22.45±3.48	0.808
***Lp(a) (mg/L)***			
Mean±S.D	180.53±13.73	238.61±9.78	0.000

## DISCUSSION

Current study shows that there was a positive relationship between risk factors of cardiovascular diseases i.e. deranged lipid profile, high blood pressure, insulin resistance and higher BMI and incidence of preeclampsia and intrauterine growth retardation. However, lipoprotein (a) was not in high concentration in patients with preeclampsia and IUGR. Recent studies have also shown that women having history of IUGR or preeclampsia have eight times higher risk of dying from cardiovascular disease when compared to the women with normal pregnancies. Therefore, researchers deduced that the risk factors leading up to cardiovascular diseases are linked to those in preeclampsia.^5^-^8^

Raised BMI, blood pressure and lipid profiles have been seen in patients with preeclampsia and these factors have also been observed in patients with cardiovascular diseases.^8^ Insulin resistance has also been observed with findings of high blood glucose and hyperinsulinemia, in patients with preeclampsia.^9^,^10^

According to a former study, there is an increased risk of cardiovascular diseases i.e. dyslipidemia, obesity, hypertension, and increased resistance to insulin in women with preeclampsia in comparison to those without any pregnancy-related complications. It is also found that in comparison to women with normal pregnancy, women with IUGR history showed higher cholesterol amounts and a risk of higher BMI, increased triglyceride amount, and increased resistance to insulin. Like outcomes are gathered in this study.^11^

A former study that evaluated the chances of the vascular dysfunction in women with IUGR or preeclampsia, suggests reduced vascular function in women with a history of IUGR without preeclampsia and early-onset preeclampsia, which can increase the chances of placental disease and increased chances of vascular diseases in future i.e. cardiovascular diseases.^12^ In women, it was concluded that in the case of previous IUGR and early-onset preeclampsia there was a significant reduction in flow-mediated vasodilation in comparison to those with previous late-onset preeclampsia and control subjects (3.2±2.7% and 2.1±1.2% versus 7.9±3.8% and 9.1±3.5%, respectively; P<0.0001).^12^ This study and previous findings show indirect relation between IUGR and preeclampsia and increased chances of cardiovascular diseases.

Women with the history of fetal IUGR have been found to have increased risk (almost 7-11 times higher) for cardiovascular diseases.^13^ This finding is not affected by the confounding variables such as smoking or socioeconomic status.

Similar findings have been reported when patients with intrauterine growth retardation are concerned. High BMI, blood pressure, triglycerides and insulin resistance syndrome have all been seen higher in patients with IUGR as well as in patients with cardiovascular diseases thus establishing a direct link between the two.^13^ Dyslipidemia is the most common risk factor that has been found to be associated with preeclampsia, IUGR and cardiovascular diseases.^13^ Lipoprotein (a) was not raised in the studied patients, however previous studies have been performed over its link to preeclampsia, IUGR and cardiovascular diseases.^14^,^15^ Local studies were performed in Pakistani population to see the total cholesterol and triglycerides in the development of preeclampsia and the results were contrary to our study.^16^,^17^

However, similar results to our study have been stated by Leerink et al.^18^ who noticed no raise in lipoprotein (a) in patients with preeclampsia or IUGR. However, Pampus et al.^19^, did state that patients with history of preeclampsia have higher concentrations of lipoprotein (a). Many other past studies also agree with the findings of the current study regarding lipoprotein (a) and preeclampsia association.^20^ Similarly Lipoprotein (a) were not raised in patients with history of IUGR. Studies have stated that as after delivery preeclampsia and IUGR condition resolves, therefore it is possible that concentrations of lipoprotein (a) returned to normal after delivery. Very few studies however have been conducted on role of lipoprotein (a) in patients with IUGR.

In summary, it can be stated that patients with history of preeclampsia tend to exhibit higher incidence of risk factors for cardiovascular diseases such as obesity, hypertension, dyslipidemia, and insulin resistance. Similarly, women with IUGR, these factors are also prevalent but to a lesser extent. However, lipoprotein (a) and triglyceride had no significance in this study as it was not raised in patients of preeclampsia or IUGR which is contrary to local study in which triglycerides has significant association with preeclampsia.^21^ These risk factors are likely to be involved in the pathogenesis of IUGR, preeclampsia and cardiovascular diseases. Thus, it is safe to say that IUGR and preeclampsia could be considered as early predictors for cardiovascular diseases.

### Limitations:

Our research has some limitations. Our study was carried out in a facility that generally serves patients from lower or middle socioeconomic classes, and the data primarily reflects the circumstance in this cohort. Smoking, socioeconomic level, and other factors that can influence the lipid profile parameters in pre-eclampsia were not taken into account in the current study.

## CONCLUSION

Findings of this study help conclude that women with known history of IUGR or preeclampsia must be screened for possible cardiovascular risk factors and treated for these risk factors in order to avoid future mortality and morbidity associated with cardiovascular diseases.

### Authors` Contribution

**FM** designed the study, wrote the protocol, data collection, and prepared manuscript.

**SA** managed the analysis and contribution in manuscript writing.

**NS and MB** managed the literature searches and review of manuscript.

**FM and SA** are responsible and accountable for the accuracy and integrity of the work.

All author’s read and approved the final manuscript. This work was carried out in collaboration among all authors.
